# A genome wide association study on Newfoundland colorectal cancer patients’ survival outcomes

**DOI:** 10.1186/s40364-015-0031-6

**Published:** 2015-03-19

**Authors:** Wei Xu, Jingxiong Xu, Konstantin Shestopaloff, Elizabeth Dicks, Jane Green, Patrick Parfrey, Roger Green, Sevtap Savas

**Affiliations:** Department of Biostatistics, Princess Margaret Cancer Centre, Toronto, ON Canada M5G 2 M9; Dalla Lana School of Public Health, University of Toronto, Toronto, ON Canada M5T 3M7; Clinical Epidemiology Unit, Faculty of Medicine, Memorial University, St. John’s, NL Canada A1B 3V6; Discipline of Genetics, Faculty of Medicine, Memorial University, St. John’s, NL Canada A1B 3 V6; Discipline of Oncology, Faculty of Medicine, Memorial University, St. John’s, NL Canada A1B 3 V6

**Keywords:** Colorectal cancer, Genome-wide, SNP, Survival analyses, Clinical outcome

## Abstract

**Background:**

In this study we performed genome-wide association studies to identify candidate SNPs that may predict the risk of disease outcome in colorectal cancer.

**Methods:**

Patient cohort consisted of 505 unrelated patients with Caucasian ancestry. Germline DNA samples were genotyped using the Illumina® human Omni-1quad SNP chip. Associations of SNPs with overall and disease free survivals were examined primarily for 431 patients with microsatellite instability-low (MSI-L) or stable (MSS) colorectal tumors using Cox proportional hazards method adjusting for clinical covariates. Bootstrap method was applied for internal validation of results. As exploratory analyses, association analyses for the colon (n = 334) and rectal (n = 171) cancer patients were also performed.

**Results:**

As a result, there was no SNP that reached the genomewide significance levels (p < 5x10^−8^) in any of the analyses. A small number of genetic markers (n = 10) showed nominal associations (p <10^−6^) for MSS/MSI-L, colon, or rectal cancer patient groups. These markers were located in two non-coding RNA genes or intergenic regions and none were amino acid substituting polymorphisms. Bootstrap analysis for the MSS/MSI-L cohort data suggested the robustness of the observed nominal associations.

**Conclusions:**

Likely due to small number of patients, our study did not identify an acceptable level of association of SNPs with outcome in MSS/MSI-L, colon, or rectal cancer patients. A number of SNPs with sub-optimal p-values were, however, identified; these loci may be promising and examined in other larger-sized patient cohorts.

**Electronic supplementary material:**

The online version of this article (doi:10.1186/s40364-015-0031-6) contains supplementary material, which is available to authorized users.

## Background

One of the current interests of medicine is to first identify and then integrate new prognostic markers in prediction models that can help distinguish cancer patients with different risks of disease outcome after diagnosis. Genetic sequence variations, such as single nucleotide polymorphisms (SNPs), may have biological roles in modifying the outcome risk and are the focus of the many current prognostic studies. Among many genetic approaches applied, the genomewide SNP survival association studies are considered useful as they examine a large number of genetic markers scattered along the DNA covering the majority of the genomic regions. Several studies applied this approach to identify genetic markers with prognostic associations in cancer such as pancreatic [1], esophageal [2], breast [3], and lung [4] cancers.

Colorectal cancer is a common malignancy [5]. Mortality rates of this disease have been decreasing in many countries including Asian [6], European [7], and North American countries (Canada [8] and the USA [9]). Yet even with this improvement in patient survival, the 5-year survival rate for this disease is estimated to be 62-64% in North America [10,11]. In other parts of the world, such as in India and Eastern European countries, these rates are lower [12]. In Canada, one of the highest incidence rates of colorectal cancer is observed in the Newfoundland population [10]. This population is also characterized by a high incidence of familial colorectal cancer [13] and by one of the lowest cancer survival rates in Canada [14].

The disease stage remains as the most important indicator of prognosis of colorectal cancer patients. Research reported in the literature suggests that other factors may also modify the prognosis, such as age at diagnosis, tumor location [15], vascular/lymphatic invasion [16], and molecular features such as the microsatellite instability (MSI) status [17,18].

MSI-high (MSI-H) is observed in almost 15% of the colon cancers and is characterized by inactivation of DNA mismatch repair genes either by germ line mutations in *MLH1*, *MSH2*, *MSH6*, and *PMS2* genes (inherited colon cancer syndrome known as the Lynch syndrome; [19]) or by the somatic promoter hypermethylation of the *MLH1* gene (sporadic colorectal cancer with MSI-H [20]). Patients with the MSI-H tumors have better survival rates than the patients with MSI-low (MSI-L) or microsatellite stable (MSS) tumors and the chromosomal instability (CIN) + tumors [17,18]. In addition, MSI-H tumor phenotype is rarely observed in rectal cancers [21] and colon and rectal cancers also differ from each other in terms of other molecular alterations, risk of recurrence, and treatment approaches [15,22,23].

Identification of genetic predictors of disease outcomes in cancer patients is a promising research aim. Till now, studies aiming to test the potential of polymorphisms as prognostic markers in colorectal cancer have been restricted to the candidate gene or pathway approaches. In this study, for the first time we performed a genomewide survival association study for patients with MSS or MSI-L tumors (MSS/MSI-L; n = 431). In addition, due to the differences between the colon and rectal cancers, as an exploratory analysis we investigated the prognostic associations of the same genetic markers in the rectal (n = 171) and colon cancer (n = 334) patients separately.

## Results

The baseline demographic, clinical and pathological data for the patients in the MSS/MSI-L, colon, and rectal cancer patient groups are shown in Table 1. The MSS/MSI-L patient group was the largest (n = 431), followed by colon (n = 334) and rectal (n = 171) patient groups. The number of events for overall survival (i.e. death) was 158, 105 and 65 in MSS/MSI-L, colon and rectal groups and for disease free survival (i.e. recurrence, metastasis or death) was 184, 121 and 79 in the same groups, respectively. For the entire cohort (n = 505), the 5-year and 10-year survival probabilities were 79.0% and 46.4% for OS and the 5-year and 10-year disease-free probabilities were 68.5% and 49.1%, respectively.

**Table 1:** **Baseline characteristics of the MSS/MSI-L colorectal, colon and rectal cancer patients investigated in this study**

**Characteristics**	**Entire cohort (n = 505)**	%*	**Colon cohort (n = 334)**	%*	**Rectal Cohort (n = 171)**	%*	**MSS/MSI-L cohort (n = 431)**	%*
Sex								
Female	198	39	141	42	57	33	158	37
Male	307	61	193	58	114	67	273	63
Age at diagnosis (median)	61.4 years, range: 20.7-75		62.25 years, range: 20.7-75		59.73 years, range: 33.64-75		62.12 years, range: 20.7-75	
Histology								
Non-mucinous	448	89	287	86	161	94	387	90
Mucinous	57	11	47	14	10	6	44	10
Location								
Colon	334	66	334	100	0	0	274	64
Rectum	171	34	0	0	171	100	157	36
Stage								
I	93	18	57	17	36	21	81	19
II	196	39	135	40	61	36	159	37
III	166	33	104	31	62	36	143	33
IV	50	10	38	11	12	7	48	11
Grade								
Well/moderately differentiated	464	92	307	92	157	92	400	93
Poorly differentiated	37	7	24	7	13	8	27	6
Unknown	4	1	3	1	1	1	4	1
Vascular invasion								
Absent	308	61	209	63	99	58	262	61
Present	159	31	107	32	52	30	137	32
Unknown	38	8	18	5	20	12	32	7
Lymphatic invasion								
Absent	298	59	200	60	98	57	256	59
Present	167	33	112	34	55	32	144	33
Unknown	40	8	22	7	18	11	31	7
Familial risk								
Low risk	250	50	161	48	89	52	218	51
High/moderate risk	255	50	173	52	82	48	213	49
MSI status								
MSS/MSI-L	431	85	274	82	157	92	431	100
MSI-H	53	10	49	15	4	2	0	0
Unknown	21	4	11	3	10	6	0	0
BRAF Val600Glu mutation								
Absent	411	81	263	79	148	87	377	87
Present	47	9	46	14	1	1	25	6
Unknown	47	9	25	7	22	13	29	7
Adjuvant chemotherapy status								
Not given	224	44	174	52	50	29	189	44
Given	277	55	156	47	121	71	239	55
Unknown	4	1	4	1	0	0	3	1
Adjuvant 5-FU based chemotherapy status								
Not given	230	46	179	54	51	30	193	45
Given	261	52	147	44	114	67	226	52
Unknown	14	3	8	2	6	4	12	3
Adjuvant radiotherapy status								
Not given	364	72	307	92	57	33	302	70
Given	124	25	11	3	113	66	113	26
Unknown	17	3	16	5	1	1	16	4
OS status								
Alive	334	66	229	69	105	61	272	63
Dead	170	34	105	31	65	38	158	37
Unknown	1	0	0	0	1	1	1	0
OS time_2010 (median)	6.36 years, range: 0.38-10.88		6.38 years, range: 0.38-10.88		6.3 years, range:1.64-10.65		6.32 years, range: 0.38-10.88	
DFS status								
No recurrence, metastasis or death	304	60	213	64	91	53	246	57
Recurrence, metastasis or death	200	40	121	36	79	46	184	43
Unknown	1	0	0	0	1	1	1	0
DFS time (median)	6 years, range: 0.22-10.88		6.22 years, range: 0.38-10.88		5.48 years, range:0.22-10.65		5.85 years, range: 0.22-10.88	

5-FU: 5-fluorouracil, DFS: disease-free survival, MSI-L: microsatellite instability-low, MSS: microsatellite stable, OS: overall survival. *numbers rounded.

The Quantile-Quantile (Q-Q) plots that were drawn for each sub-group and each outcome are shown in the Additional file 1. These plots show that the models on the genome-wide scan satisfy the expected distribution and suggest the appropriateness of the multivariate model settings.

As a result of the statistical analysis, possibly due to the limited sample size, no association with genome-wide association significance levels (p < 5.0E-08) was detected in models constructed. But, ten SNPs showed suggestive associations with OS and DFS times (p < 1.0E-06), which is the nominal significance cut-off level in our study (Table 2). Manhattan plots for the patient cohorts and outcomes are shown in Additional file 1. For the interested readers, the list of associations detected at a p-value less than 1.0E-05 can be found in Additional file 2.

**Table 2:** **SNPs identified from six models with nominal significance (p < 1.0x10** ^**−6**^ **)**

**Group/Outcome**	**Gene**	**SNP**	**Chr**	**Position**	**MAF**	**HWE-p**	**genotype**	**p-value**	**HR**	**CI_low**	**CI_high**
MSS/MSI-L-DFS	LINC01121	rs6720296	2	45408269	0.4026	0.48	150 215 66	5.46E-07	1.74	1.40	2.16
MSS/MSI-L-DFS	n/a (intergenic)	rs1407508	9	101644538	0.0580	0.65	383 46 2	8.51E-07	2.53	1.75	3.66
colon-OS	n/a (intergenic)	rs4812219	20	59422971	0.0823	1.00	281 51 2	7.41E-07	3.27	2.05	5.23
colon-DFS	n/a (intergenic)	rs17280262	14	97053924	0.0629	0.13	295 36 3	1.33E-07	3.02	2.00	4.55
rectum-OS	n/a (intergenic)	rs17026425	4	150672514	0.0439	0.27	157 13 1	6.65E-07	5.06	2.67	9.60
rectum-OS	n/a (intergenic)	rs6854845	4	75746665	0.1082	0.70	135 35 1	9.46E-07	4.12	2.34	7.26
rectum-DFS	n/a (intergenic)	rs1570271	10	115288501	0.0882	1.00	141 28 1	1.47E-07	3.66	2.26	5.94
rectum-DFS	AC011343.1	rs17057166	5	159248014	0.0468	1.00	155 16 0	2.11E-07	5.56	2.91	10.64
rectum-DFS	n/a (intergenic)	rs4868304	5	173131457	0.1550	0.25	124 41 6	2.52E-07	2.91	1.94	4.37
rectum-DFS	n/a (intergenic)	rs6854845	4	75746665	0.1082	0.70	135 35 1	6.16E-07	3.31	2.07	5.30

Chr: chromosome, CI_high: higher bound of the 95% confidence interval for the HR estimate, CI_low: lower bound of 95% confidence interval for the HR estimate; DFS: disease-free survival, HWE-p: p-value for the Hardy-Weinberg Equilibrium test, HR: hazards ratio, MAF: minor allele frequency, MSS: microsatellite stable, MSI-L: microsatellite instability-low, OS: overall survival, SNP: single nucleotide polymorphism. Please note that the genotyping platform also contain other types of genetic markers, such as small insertions/deletions. For simplicity, all genetic markers are annotated as SNPs throughout this manuscript; interested readers may check the dbSNP database ([24]) for further information on these SNPs. Gene information as noted in the dbSNP database for each of the SNPs.

In the MSS/MSI-L patient group association study, there were two SNPs in the DFS model that achieved the nominal-significance level (Table 2). The HRs obtained as a result of the bootstrap method demonstrated the robustness of the results from the original association analysis (Additional file 3). One of these SNPs (rs6720296) was a frequent variant (MAF: 40%) and based on the information in the dbSNP database [24], was located in a non-coding RNA gene (long intergenic non-protein coding RNA 1121; LINC01121). The second marker (rs1407508) was a relatively infrequent SNP (MAF: 5.8%) located in a non-coding region on chromosome 9.

In the colon patient group analysis, two SNPs showed nominally-significant signals in OS or DFS models (Table 2). These SNPs were located in non-coding regions along the chromosome 20 and 14.

Among all groups, the rectal cancer patient group was the one with the largest group of SNPs identified, the majority of which were intergenic. In this patient cohort, two SNPs in OS model and four SNPs in DFS model had p-value lower than the cut-off level (p < 1.0x10^−6^) (Table 2). Interestingly, one intergenic SNP was associated with both OS and DFS in this patient group (rs6854845). Among the six SNPs, rs17057166 was the only one located in a gene (non-coding RNA; AC011343.1).

None of the SNPs in Table 2 were amino acid changing SNPs (non-synonymous). As of February 2015, there was no publication in PUBMED about these SNPs. A search at the Regulome DB database [25] returned information for five of the polymorphisms (rs17026425, rs1407508, rs17280262, rs17057166, and rs6854845). According to these results, rs17026425 (Regulome DB score: 3a) is located within a transcription factor (such as JUND) binding site, yet it is “relatively less likely to affect protein binding”. For rs1407508 (Regulome DB score: 4), rs17280262, rs17057166, and rs6854845 (Regulome DB scores: 5), Regulome DB suggests that there is minimal or no evidence of proteins binding to the DNA sequences where these SNPs are located.

An additional information related to the genes for the SNPs with p-values less than 1.0x10^−5^ is shown in the Additional file 4.

## Discussion

In this study, we aimed to investigate the associations of a large number of SNPs (n = 729,737) with overall or disease free survival times in Caucasian colorectal cancer patients from Newfoundland, Canada. Our primary aim was to investigate the associations of SNPs with the disease outcome in the patients with MSI-L or MSS tumors (n = 431) as the outcome risks for this group of patients and the patients with the MSI-H tumors are significantly different from each other [17,18]. In addition, due to the differences between the colon and rectal cancer patients (for example the relatively decreased recurrence risk as well as the higher number of MSI-H tumors in colon cancer patients compared to the rectal cancer patients [15,21]), as an exploratory analysis, separate analyses for the colon (n = 334) and rectal (n = 171) cancer patients were also performed. Of note, due to the small number of the patients with MSI-H tumors (n = 53), we have not attempted the statistical analyses in this group.

The main result of this study is that none of the genetic markers investigated reached the genomewide significance levels (5.0E-08) in either overall or disease free survival analyses in any of the patient groups investigated. Thus, we were not able to identify a genetic marker with a strong association with the risk of main clinical outcomes (i.e. death, local or distant metastases) in colorectal cancer. This can be interpreted as that none of the genetic markers investigated are related to the survival outcomes of interest. Alternatively, this can be also due to the fact that the sample sizes of the patient cohorts investigated in this study were small and thus our study power was limited to detect possible associations (see Methods). Considering this study power issue, in this manuscript we present and discuss the SNPs with p-values (<1.0E-06) higher than the genome-wide significance levels as potentially promising genetic markers (Table 2). We suggest that these markers may be promising and should be investigated in larger-sized cohorts to test whether they are associated with the colorectal cancer prognosis. Further research may also be performed to test whether the two non-coding RNA genes (AC011343.1 and LINC01121) shown in Table 2 have prognostic roles in colorectal cancer. In addition, while there is currently no literature report about the SNPs in Table 2 showing their biological functions or relation to health and disease, Regulome DB [25] data suggest that one of the SNPs, rs17026425, is located within the binding site of JUN/JUND transcription factors. Mammalian JUN family of transcriptional regulators includes c-JUN, JUNB, and JUND with important roles in cell proliferation and carcinogenesis (reviewed in [26]). According to our results, rs17026425 polymorphism was nominally associated with overall survival in rectal cancer sub-group (Table 2). Further studies on rs17026425 polymorphism may test its potential binding to the JUN family of transcription factors, its potential role in variable JUN function, and contribution to the rectal cancer formation and progression.

## Conclusions

In conclusion, we performed genomewide SNP survival association studies in MSS/MSI-L, colon and rectal cancer patients. A limitation of this study is that all three patient cohorts investigated were characterized by small samples sizes, thus we cannot confidently conclude whether the investigated genetic markers indeed have no prognostic associations in colorectal cancer. However, this study also generated a small set of SNPs that may be an interest for other investigators in their future analyses.

## Methods

### Patient cohort

Patients registered at the Newfoundland Colorectal Cancer Registry (NFCCR) were investigated in the present study. Characteristics of the NFCCR patient cohort were described earlier [13,27]. Briefly, between 1999–2003, a total of 750 participants were recruited to NFCCR. Informed consent was obtained from the patients or their family members. Recurrence, metastasis or vital status information for patients were collected till 2010 as described in Negandhi et al. [28]. Tumor characteristics (i.e. MSI status) were determined as explained by Woods et al. [27]. Among 750 patients, 736 stage I-IV patients had the prognostic data collected during the follow-up period.

### Genotyping and quality control (QC)

Germline DNA was isolated from patients’ blood samples. Initially, a total of 539 patients with available prognostic data and germline DNA were subject to whole-genome SNP genotyping using the Illumina® Omni1-Quad human SNP genotyping platform (service provider: Centrillion Biosciences, USA).

QC analyses on the genetic data were performed using PLINK v1.07 [29] and Eigensoft 4.2 [30]. We excluded 1) one subject because of mismatching sex information; 2) 129,172 SNPs with high missing genotype data (above 5%); 3) 21 individuals who were first, second, or third degree relatives (based on the identity by state PI_HAT score >0.125), and 4) one subject with extreme value of heterozygosity (out of 6 standard deviations). Additionally, Multidimensional-scaling (MDS) method was used to identify individuals with diversity in ancestry. Comparing with the Hapmap III points in the MDS plot, six outliers that are far away from Caucasian cluster were removed from the study (Additional file 5). Lastly, 275,285 SNPs and 320 SNPs were excluded because of failure in HWE test (p < 1.0E-08) and low minor allele frequency (MAF < 0.05), respectively.

Principal component analysis was undertaken with the EIGENSTRAT package after QC filtering (Additional file 5). The first two principal components were incorporated into the models. Additional analyses for population stratification were undertaken with each of the genetic marker adjusting the estimated principal components. As a result, five population outliers were identified and excluded from the analysis.

After these analyses, a total of 505 subjects and 729,737 SNPs were included in the final analysis. The adequacy of the probability distribution and possibility of differential genotyping were formally evaluated using the Q-Q plots of –log10(p-values) (Additional file 1).

### Association analyses

We examined the associations of SNPs with two major clinical outcomes: overall survival (OS) and disease free survival (DFS). We define OS as the date from diagnosis till the date of death from any cause or the date of last follow-up, and DFS as the date from diagnosis till the date of local recurrence, metastasis or death from any cause, or the date of last follow-up. OS or DFS status was available for 504 patients.

Cox proportional hazard models were applied based on a genetic additive model. Hazard Ratio (HR) estimates and corresponding 95% confidence intervals (CIs) were calculated using R statistical programming language [31]. The association between genetic markers and survival outcomes, OS and DFS, adjusting for the top two principal components and other confounding factors (gender and stage for MSI-L or MSS sub-group, gender, stage and MSI for colon and rectal sub-groups), was tested using Cox proportional hazard model.

Clinical factors, as potential confounding factors in the genetic analysis, were evaluated firstly using univariate analysis. Among all the clinical factors: gender, stage, MSI were found to be significantly associated with OS (p-values: 0.0151, 2.2E-16, 5.2E-05, respectively); and gender, site (colon/rectum), stage, MSI were shown to be significantly associated with DFS (p-values: 0.0139, 0.0344, 3.3E-16, 1.2E-04, respectively). These clinical variables were used to build up baseline multivariable models for OS and DFS separately in each sub-cohort.

In order to present the genetic association with the outcome variable in more detail, MSS/MSI-L patient, colon cancer patients and rectal cancer patient groups were analysed separately using Cox proportional hazards model. Since two outcome measures (overall and disease free survivals) were examined in MSS/MSI-L, colon and rectum cancer patient cohorts, a total of six models were constructed. While for the genome-wide screening the genetic markers were modeled using additive genetic models, dominant, recessive, and co-dominant genetic models were also applied as sensitivity analysis to assess the genetic inheritance effect. For each scenario, two types of models were explored, namely crude, and comprehensive models: for the crude model, we adjusted by principal components only; for the comprehensive model, we adjusted by principal components and the baseline model factors. In this report we show the comprehensive models.

In order to validate the results obtained from the MSS/MSI-L cohort association study, we applied bootstrap resample method. Based on the original data set, 200 bootstrap samples data sets were generated using sampling with replacement algorithm. For each of these bootstrap data sets, we applied Cox proportional hazards regression model on the SNPs with significance levels p < 1.0x10^−5^ identified from the original analysis and calculated the corresponding HRs of the genetic effect. Overall, 200 HRs were provided for each SNP and bootstrap confidence intervals were created.

We computed the study power for the MSS-MSI-L cohort (n = 431) using PASS 13 (NCSS, LLC. Kaysville, Utah, USA). Assuming a SNP in linkage disequilibrium, LD, (D’ = 1) with a risk allele frequency 0.3, we have 0.76 power to detect nominal significant association at p < 1.0x10^−6^ under a dominant model with strong effect size of HR 2.0 in the MSS-MSI-L cohort. To detect an association with the same assumptions and at p < 5E-08 significance level, the statistical power is reduced to 0.56. With a moderate effect size of HR 1.5, the power to detect genome wide significant association (p < 5E-08) is very low (0.08).

## Additional files

Additional file 1:
**Q-Q and Manhattan plots.** a) OS analysis in MSS/MSI-L patient sub-cohort (Inflation factor: 1.024), b) DFS analysis in MSS/MSI-L patient sub-cohort (Inflation factor: 0.971), c) OS analysis in colon cancer patient sub-cohort (Inflation factor: 1.015), d) DFS analysis in colon cancer patient sub-cohort (Inflation factor: 0.958), e) OS analysis in rectal cancer patient sub-cohort (Inflation factor: 1.042), f) DFS analysis in rectal cancer patient sub-cohort (Inflation factor: 1.005). Additional file 2:
**SNPs identified from six models with significance values of p < 10** ^**-5**^ **.**
Additional file 3:
**Hazard ratios obtained by the bootstrap method for the MSS/MSI-L patient cohort.**
Additional file 4:
**Information on the genic locations of the genetic polymorphisms with p < 1.0x10** ^**-5**^ **.**
Additional file 5:
**Principal Component Analysis (PCA) and Multidimensional Scaling (MDS) analysis results for the patient cohort.**

## Abbreviations

CI: Confidence interval
CIN: Chromosomal instability
DFS: Disease-free survival
HR: Hazards ratio
LD: Linkage disequilibrium
MAF: Minor allele frequency
MDS: Multidimensional scaling
MSI: Microsatellite instability
MSI-H: Microsatellite instability-high
MSS: Microsatellite stable
NFCCR: Newfoundland Colorectal Cancer Registry
OS: Overall survival
PCA: Principal component analysis
SNP: Single nucleotide polymorphism
QC: Quality control
