# Supplementary material for: A genome wide association study on Newfoundland colorectal cancer patients’ survival outcomes
Source: Biomark Res. 2015 Mar 19;3:6. doi: 10.1186/s40364-015-0031-6 (PMC4393623; doi:10.1186/s40364-015-0031-6)
Supplement: Additional file 1: — Q-Q and Manhattan plots. a) OS analysis in MSS/MSI-L patient sub-cohort (Inflation factor: 1.024), b) DFS analysis in MSS/MSI-L patient sub-cohort (Inflation factor: 0.971), c) OS analysis in colon cancer patient sub-cohort (Inflation factor: 1.015), d) DFS analysis in colon cancer patient sub-cohort (Inflation factor: 0.958), e) OS analysis in rectal cancer patient sub-cohort (Inflation factor: 1.042), f) DFS analysis in rectal cancer patient sub-cohort (Inflation factor: 1.005). [file 40364_2015_31_MOESM1_ESM.pdf]

**Additional File 1.** Q-Q and Manhattan plots

a) OS analysis in MSS/MSI-L patient sub-cohort (Inflation factor: 1.024)

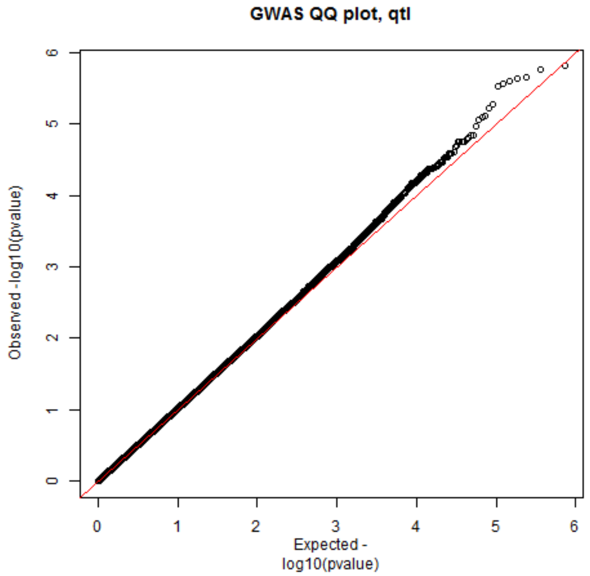


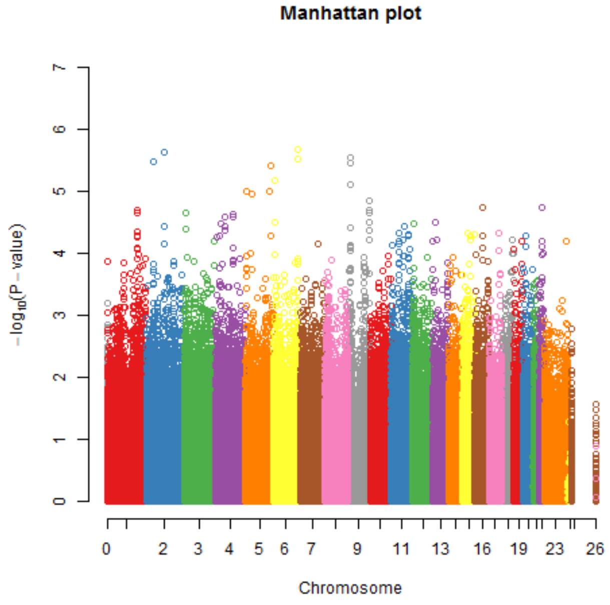

b) DFS analysis in MSS/MSI-L patient sub-cohort (Inflation factor: 0.971)

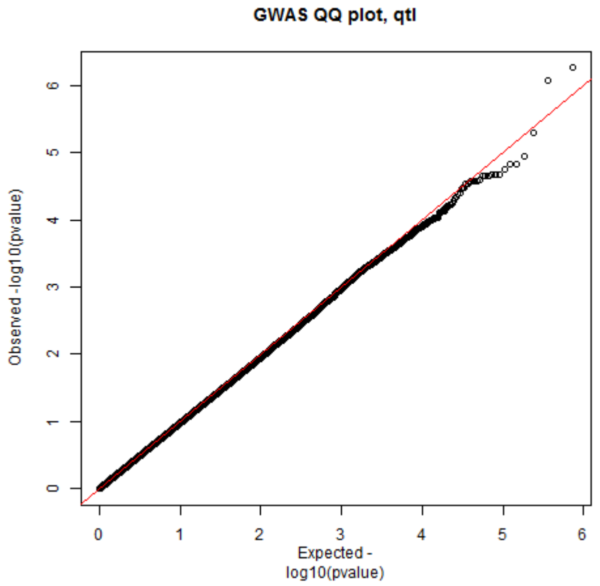


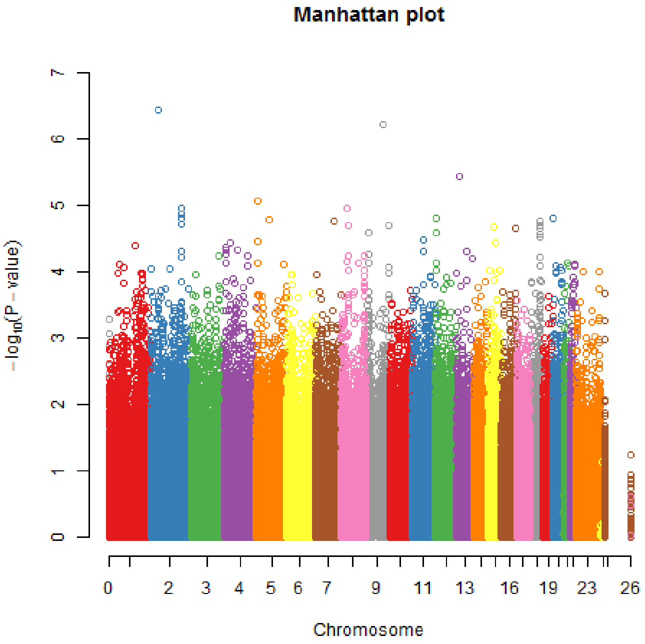

c) OS analysis in colon cancer patient sub-cohort (Inflation factor: 1.015)

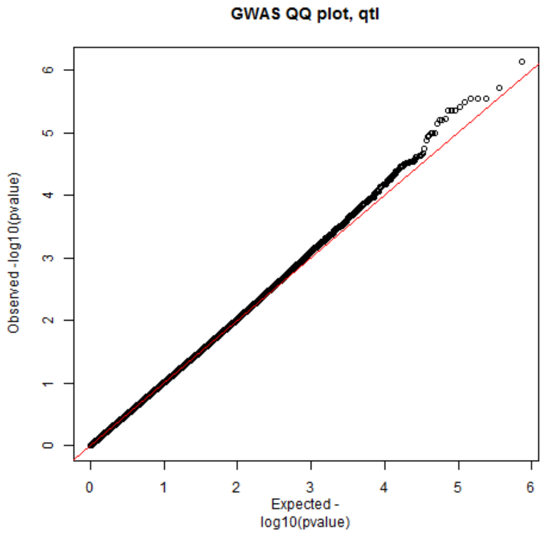


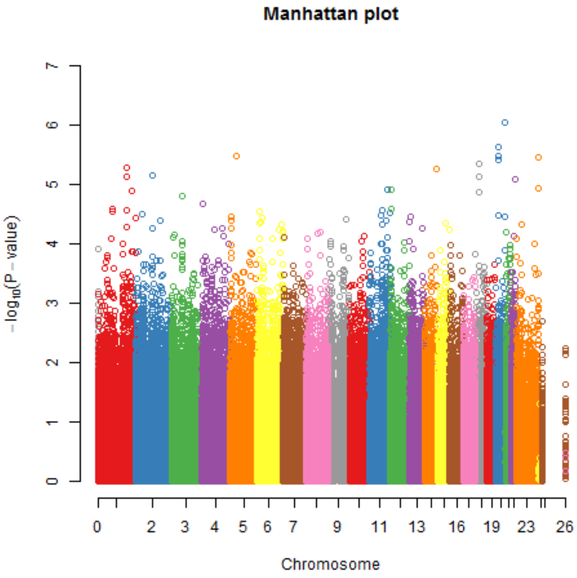

d) DFS analysis in colon cancer patient sub-cohort (Inflation factor: 0.958)

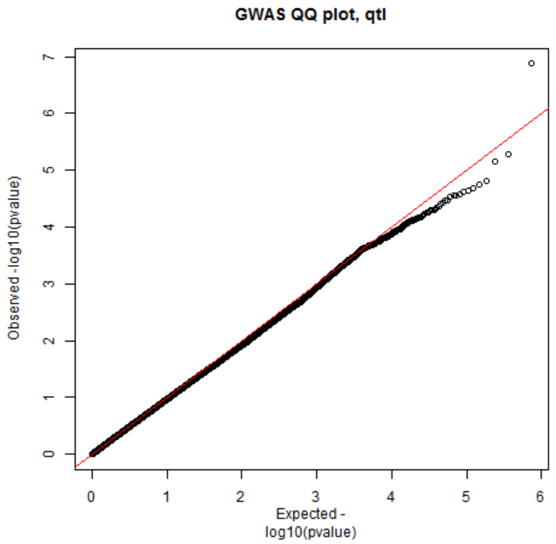


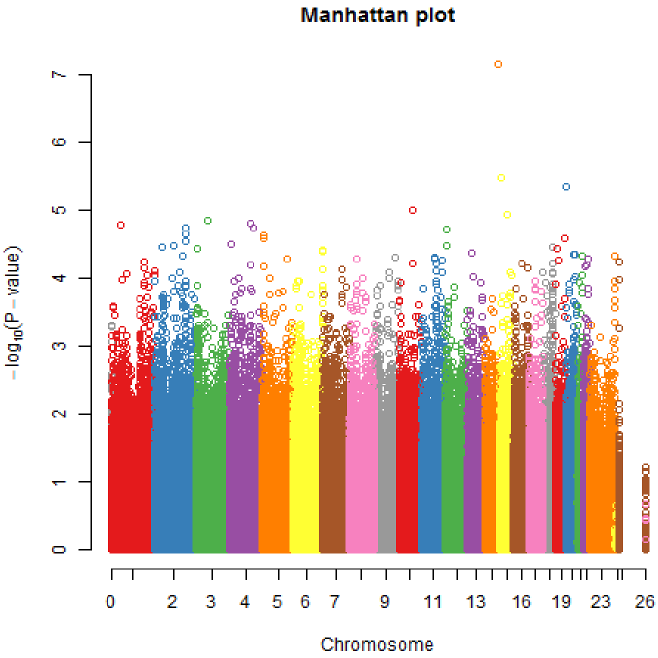

e) OS analysis in rectal cancer patient sub-cohort (Inflation factor: 1.042)

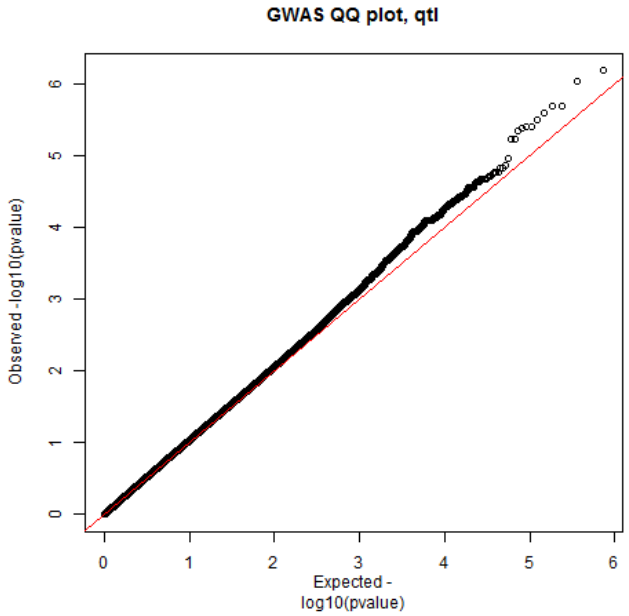


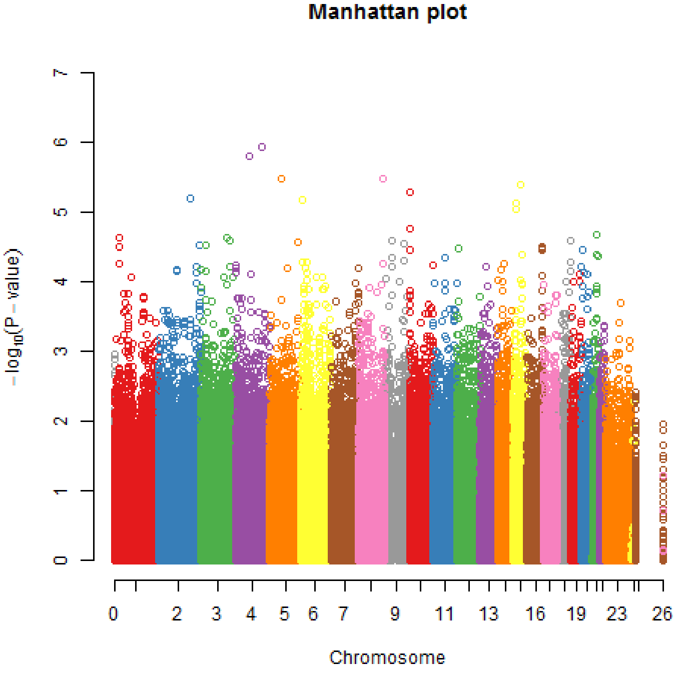

f) DFS analysis in rectal cancer patient sub-cohort (Inflation factor: 1.005)

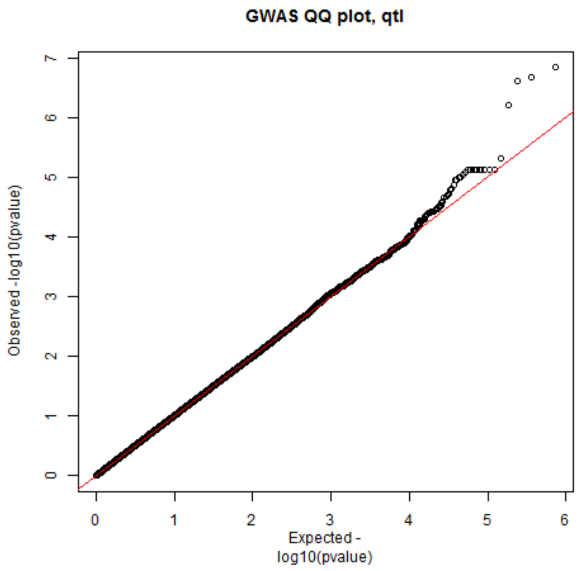


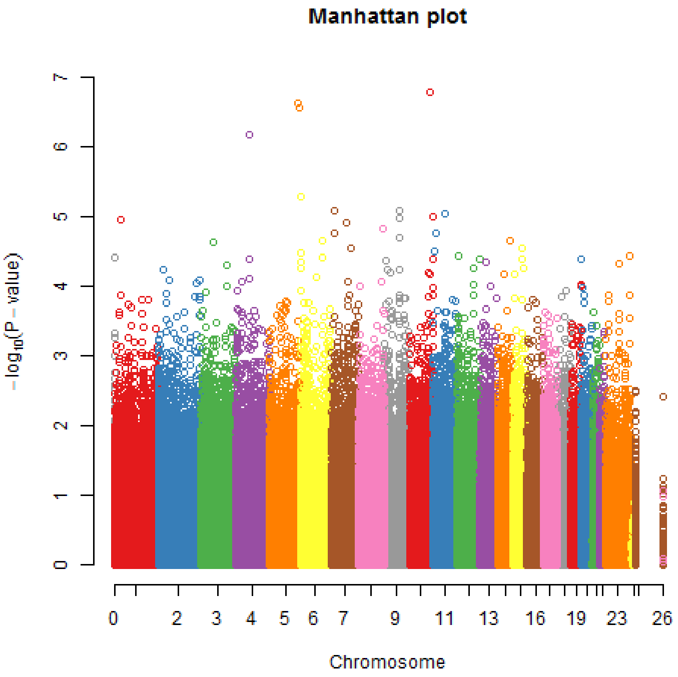


The Q-Q plots show that the models on the genome scan satisfy the expected distribution, and suggest the appropriate of the multivariate model setting. However, none of the Manhattan plot

shows genome wide significant signals. It may due to limited sample size or moderate effect size of the genetic association.
