# Supplementary material for: A genome wide association study on Newfoundland colorectal cancer patients’ survival outcomes
Source: Biomark Res. 2015 Mar 19;3:6. doi: 10.1186/s40364-015-0031-6 (PMC4393623; doi:10.1186/s40364-015-0031-6)
Supplement: Additional file 2: — SNPs identified from six models with significance values of p < 10 -5 . [file 40364_2015_31_MOESM2_ESM.pdf]

Additional File 2. SNPs identified from six models with nominal significance ($p<10^{-5}$)

Group/Outcome	Gene	SNP	Chr	Position	MAF	HWE-p	genotype	p-value	HR	CI_low	CI_high
MSS/MSI-L-OS	LOC101928923	rs17087282	6	156588020	0.0580	0.16	384 44 3	1.55E-06	2.47	1.71	3.56
MSS/MSI-L-OS	DPP10	rs17048372	2	115213756	0.2088	0.08	276 130 25	1.71E-06	1.91	1.46	2.48
MSS/MSI-L-OS	n/a (intergenic)	rs1998584	9	13704539	0.4512	0.70	127 218 85	2.18E-06	1.76	1.39	2.22
MSS/MSI-L-OS	LOC101928923	rs6917119	6	156585838	0.0754	0.03	372 53 6	2.31E-06	2.15	1.57	2.96
MSS/MSI-L-OS	LINC01121	rs6720296	2	45408269	0.4026	0.48	150 215 66	2.50E-06	1.74	1.38	2.19
MSS/MSI-L-OS	n/a (intergenic)	rs992457	9	13705882	0.4314	0.77	137 215 78	2.68E-06	1.74	1.38	2.19
MSS/MSI-L-OS	n/a (intergenic)	rs12187751	5	162585645	0.0789	0.50	364 66 1	2.94E-06	2.48	1.70	3.64
MSS/MSI-L-OS	n/a (intergenic)	rs1573948	6	6786425	0.1311	0.52	327 95 9	5.21E-06	1.93	1.46	2.57
MSS/MSI-L-OS	n/a (intergenic)	rs1590404	9	13697152	0.4385	0.63	133 218 80	5.88E-06	1.71	1.35	2.15
MSS/MSI-L-OS	n/a (intergenic)	rs10040610	5	6353815	0.1221	0.50	333 89 8	7.73E-06	1.96	1.46	2.63
MSS/MSI-L-OS	GRIA1	rs1493383	5	152991398	0.1787	0.63	292 124 15	7.86E-06	1.81	1.39	2.35
MSS/MSI-L-OS	HCN1	rs13180087	5	45265768	0.1195	0.65	335 89 7	8.74E-06	2.06	1.50	2.83
**MSS/MSI-L-DFS**	**LINC01121**	**rs6720296**	**2**	**45408269**	**0.4026**	**0.48**	**150 215 66**	**5.46E-07**	**1.74**	**1.40**	**2.16**
**MSS/MSI-L-DFS**	**n/a (intergenic)**	**rs1407508**	**9**	**101644538**	**0.0580**	**0.65**	**383 46 2**	**8.51E-07**	**2.53**	**1.75**	**3.66**
MSS/MSI-L-DFS	n/a (intergenic)	rs912294	13	31698294	0.4594	0.10	117 232 82	5.07E-06	1.67	1.34	2.08
**colon-OS**	**n/a (intergenic)**	**rs4812219**	**20**	**59422971**	**0.0823**	**1.00**	**281 51 2**	**7.41E-07**	**3.27**	**2.05**	**5.23**
colon-OS	ISM1	rs6105057	20	13229326	0.2231	0.27	205 109 20	1.93E-06	2.20	1.59	3.04
colon-OS	ISM1	rs6134830	20	13225350	0.2184	0.20	207 105 20	2.81E-06	2.17	1.57	3.01
colon-OS	HCN1	rs13180087	5	45265768	0.1243	0.62	257 71 6	2.82E-06	2.40	1.66	3.46

colon-OS	n/a (intergenic)	rs17325431	23	148462072	0.0524	0.00	311 11 12	2.87E-06	2.47	1.69	3.61
colon-OS	ISM1	rs6109769	20	13216257	0.2216	0.43	205 110 19	3.18E-06	2.19	1.57	3.04
colon-OS	DSG3	rs3794924	18	29041734	0.1123	0.40	261 71 2	3.77E-06	2.37	1.65	3.43
colon-OS	n/a (intergenic)	rs10921219	1	192587863	0.4192	0.74	111 166 57	4.32E-06	1.90	1.44	2.49
colon-OS	n/a (intergenic)	rs10733072	1	192596512	0.4192	0.74	111 166 57	4.32E-06	1.90	1.44	2.49
colon-OS	n/a (intergenic)	rs17280262	14	97053924	0.0629	0.13	295 36 3	4.49E-06	2.66	1.75	4.04
colon-OS	DPP10	rs17048372	2	115213756	0.2111	0.32	211 105 18	5.88E-06	2.17	1.55	3.03
colon-OS	DSG3	rs8091481	18	29051677	0.1153	0.28	259 73 2	6.17E-06	2.33	1.61	3.36
colon-OS	n/a (intergenic)	rs12062810	1	192569804	0.4159	0.82	112 165 56	6.30E-06	1.88	1.43	2.48
colon-OS	PARVG	rs139156	22	44596085	0.0793	0.45	284 47 3	6.98E-06	2.76	1.77	4.29
colon-OS	n/a (intergenic)	rs4844011	23	148392640	0.0509	0.00	311 12 11	9.91E-06	2.40	1.63	3.54
**colon-DFS**	**n/a (intergenic)**	**rs17280262**	**14**	**97053924**	**0.0629**	**0.13**	**295 36 3**	**1.33E-07**	**3.02**	**2.00**	**4.55**
colon-DFS	n/a (intergenic)	rs8035094	15	33564482	0.0674	0.05	293 37 4	5.23E-06	2.68	1.75	4.09
colon-DFS	C20orf27	rs658495	20	3737495	0.0554	0.26	299 33 2	7.08E-06	2.53	1.69	3.78
**rectum-OS**	**n/a (intergenic)**	**rs17026425**	**4**	**150672514**	**0.0439**	**0.27**	**157 13 1**	**6.65E-07**	**5.06**	**2.67**	**9.60**
**rectum-OS**	**n/a (intergenic)**	**rs6854845**	**4**	**75746665**	**0.1082**	**0.70**	**135 35 1**	**9.46E-07**	**4.12**	**2.34**	**7.26**
rectum-OS	n/a (intergenic)	rs157411	5	67294907	0.2398	1.00	99 62 10	2.02E-06	2.89	1.86	4.47
rectum-OS	EFR3A	rs7004484	8	132934538	0.2164	1.00	105 58 8	2.03E-06	2.79	1.83	4.25
rectum-OS	n/a (intergenic)	rs338389	15	68260008	0.5000	0.05	36 99 36	2.53E-06	3.40	2.04	5.67
rectum-OS	n/a (intergenic)	rs1555895	10	837407	0.4678	0.22	44 94 33	3.16E-06	0.34	0.22	0.54
rectum-OS	n/a (intergenic)	rs16867335	2	181458934	0.1871	0.80	112 54 5	3.99E-06	3.27	1.98	5.42
rectum-OS	n/a (intergenic)	rs6739798	2	181465853	0.1871	0.80	112 54 5	3.99E-06	3.27	1.98	5.42
rectum-OS	n/a (intergenic)	rs1573948	6	6786425	0.0936	0.16	142 26 3	4.10E-06	2.71	1.77	4.15

rectum-OS	n/a (intergenic)	rs10152207	15	38129779	0.1170	0.06	136 30 5	4.55E-06	3.06	1.90	4.93
rectum-OS	n/a (intergenic)	rs1827439	15	38123182	0.1111	0.04	138 28 5	5.84E-06	3.06	1.89	4.96
rectum-OS	n/a (intergenic)	rs10153021	15	38123785	0.1111	0.04	138 28 5	5.84E-06	3.06	1.89	4.96
**rectum-DFS**	**n/a (intergenic)**	**rs1570271**	**10**	**115288501**	**0.0882**	**1.00**	**141 28 1**	**1.47E-07**	**3.66**	**2.26**	**5.94**
**rectum-DFS**	**AC011343.1**	**rs17057166**	**5**	**159248014**	**0.0468**	**1.00**	**155 16 0**	**2.11E-07**	**5.56**	**2.91**	**10.64**
**rectum-DFS**	**n/a (intergenic)**	**rs4868304**	**5**	**173131457**	**0.1550**	**0.25**	**124 41 6**	**2.52E-07**	**2.91**	**1.94**	**4.37**
**rectum-DFS**	**n/a (intergenic)**	**rs6854845**	**4**	**75746665**	**0.1082**	**0.70**	**135 35 1**	**6.16E-07**	**3.31**	**2.07**	**5.30**
rectum-DFS	SLC22A23	rs4959799	6	3295028	0.0673	0.55	149 21 1	4.84E-06	3.50	2.05	5.99
rectum-DFS	n/a (intergenic)	rs11138220	9	82060528	0.0760	0.06	148 20 3	7.59E-06	2.76	1.77	4.31
rectum-DFS	n/a (intergenic)	rs11138231	9	82066164	0.0760	0.06	148 20 3	7.59E-06	2.76	1.77	4.31
rectum-DFS	n/a (intergenic)	rs1015311	9	82078454	0.0760	0.06	148 20 3	7.59E-06	2.76	1.77	4.31
rectum-DFS	n/a (intergenic)	rs11138250	9	82083082	0.0760	0.06	148 20 3	7.59E-06	2.76	1.77	4.31
rectum-DFS	n/a (intergenic)	rs10491791	9	82096194	0.0760	0.06	148 20 3	7.59E-06	2.76	1.77	4.31
rectum-DFS	n/a (intergenic)	rs13286432	9	82098952	0.0760	0.06	148 20 3	7.59E-06	2.76	1.77	4.31
rectum-DFS	n/a (intergenic)	rs1890836	9	82119180	0.0760	0.06	148 20 3	7.59E-06	2.76	1.77	4.31
rectum-DFS	*n/a (intergenic)	rs10275272	7	19160897	0.1053	0.22	135 36 0	7.60E-06	3.19	1.92	5.31
rectum-DFS	ANO1	rs3781663	11	69999252	0.2982	0.15	80 80 11	8.35E-06	2.24	1.57	3.19
rectum-DFS	n/a (intergenic)	rs9419702	10	133531153	0.2105	0.82	107 56 8	9.24E-06	2.54	1.68	3.83
rectum-DFS	n/a (intergenic)	rs11138252	9	82087111	0.0912	0.14	142 25 3	9.95E-06	2.68	1.73	4.14

Chr: chromosome, CI_high: higher bound of the 95% confidence interval for the HR estimate, CI_low: lower bound of 95% confidence interval for the HR estimate; DFS: disease-free survival, HWE-p: p-value for the Hardy-Weinberg Equilibrium test, HR:

hazards ratio, MAF: minor allele frequency, MSS: microsatellite stable, MSI-L: microsatellite instability-low, OS: overall survival, SNP: single nucleotide polymorphism. Please note that the genotyping platform also contain other types of genetic markers, such as small insertions/deletions. For simplicity, all genetic markers are annotated as SNPs throughout this manuscript; interested readers may check the dbSNP database (1) for further information on these SNPs. Gene information as noted in the dbSNP database for each of the SNPs. The SNPs with p-values less than $1.0x10^{-6}$ are in bold.
