# Supplementary material for: A genome wide association study on Newfoundland colorectal cancer patients’ survival outcomes
Source: Biomark Res. 2015 Mar 19;3:6. doi: 10.1186/s40364-015-0031-6 (PMC4393623; doi:10.1186/s40364-015-0031-6)
Supplement: Additional file 3: — Hazard ratios obtained by the bootstrap method for the MSS/MSI-L patient cohort. [file 40364_2015_31_MOESM3_ESM.pdf]

Additional File 3. Hazard ratios obtained by the bootstrap method for the MSS/MSI-L patient cohort

		Discovery			Bootstrap validation		
Group/outcome	SNP	HR	CI_low	CI_high	HR	CI_low	CI_high
MSS/MSI-L-OS	rs17087282	2.465903	1.706457	3.563334	2.489347	1.589483	3.389212
MSS/MSI-L-OS	rs17048372	1.907287	1.464073	2.484674	1.937508	1.406002	2.469015
MSS/MSI-L-OS	rs1998584	1.755761	1.390864	2.216391	1.772483	1.323602	2.221364
MSS/MSI-L-OS	rs6917119	2.153151	1.566343	2.959798	2.206778	1.357627	3.055928
MSS/MSI-L-OS	rs6720296	1.73673	1.380191	2.185372	1.750713	1.375104	2.126322
MSS/MSI-L-OS	rs992457	1.737071	1.379346	2.18757	1.753912	1.329533	2.17829
MSS/MSI-L-OS	rs12187751	2.48452	1.696405	3.638776	2.583014	1.494313	3.671716
MSS/MSI-L-OS	rs1573948	1.934566	1.456474	2.569592	1.979115	1.352131	2.606099
MSS/MSI-L-OS	rs1590404	1.705313	1.353708	2.148241	1.725509	1.295729	2.15529
MSS/MSI-L-OS	rs10040610	1.960098	1.459453	2.632482	2.009739	1.38067	2.638808
MSS/MSI-L-OS	rs1493383	1.809274	1.394982	2.346606	1.831101	1.353747	2.308454
MSS/MSI-L-OS	rs13180087	2.056061	1.496372	2.82509	2.113158	1.397274	2.829043
MSS/MSI-L-DFS	rs6720296	1.73848	1.400231	2.158439	1.764961	1.41425	2.115673
MSS/MSI-L-DFS	rs1407508	2.527202	1.747212	3.655395	2.634727	1.45264	3.816814
MSS/MSI-L-DFS	rs912294	1.667675	1.338686	2.077516	1.682737	1.310622	2.054852

CI: confidence interval, CI_high: higher bound of the 95% confidence interval for the HR estimate, CI_low: lower bound of 95% confidence interval for the HR estimate; DFS: disease-free survival, HR: hazards ratio, MSS: microsatellite stable, MSI-L: microsatellite instability-low, OS: overall survival, SNP: rs numbers for the single nucleotide polymorphism.
