# Supplementary material for: A genome wide association study on Newfoundland colorectal cancer patients’ survival outcomes
Source: Biomark Res. 2015 Mar 19;3:6. doi: 10.1186/s40364-015-0031-6 (PMC4393623; doi:10.1186/s40364-015-0031-6)
Supplement: Additional file 4: — Information on the genic locations of the genetic polymorphisms with p < 1.0x10 -5 . [file 40364_2015_31_MOESM4_ESM.pdf]

Additional File 4. Information on the genic locations of the genetic polymorphisms nominally ($p<1.0x10^{-5}$) associated with the outcomes.

Group/Outcome	Locus (dbSNP (1))	Gene name (Entrez Gene (2))	Gene ID (Entrez Gene (2))	Gene type (Entrez Gene (2))	SNP	Chr	Position
MSS/MSI-L-OS	LOC101928923	LOC101928923	101928923	non-coding RNA (uncharacterized)	rs17087282	6	156588020
MSS/MSI-L-OS	DPP10	dipeptidyl-peptidase 10	57628	protein-coding (non-functional)	rs17048372	2	115213756
MSS/MSI-L-OS	n/a (intergenic)	n/a	n/a	n/a	rs1998584	9	13704539
MSS/MSI-L-OS	LOC101928923	uncharacterized LOC101928923	101928923	non-coding RNA (uncharacterized)	rs6917119	6	156585838
MSS/MSI-L-OS	LINC01121	long intergenic non-protein coding RNA 1121	400952	non-coding RNA	rs6720296	2	45408269
MSS/MSI-L-OS	n/a (intergenic)	n/a	n/a	n/a	rs992457	9	13705882
MSS/MSI-L-OS	n/a (intergenic)	n/a	n/a	n/a	rs12187751	5	162585645
MSS/MSI-L-OS	n/a (intergenic)	n/a	n/a	n/a	rs1573948	6	6786425
MSS/MSI-L-OS	n/a (intergenic)	n/a	n/a	n/a	rs1590404	9	13697152
MSS/MSI-L-OS	n/a (intergenic)	n/a	n/a	n/a	rs10040610	5	6353815
MSS/MSI-L-OS	GRIA1	glutamate receptor, ionotropic, AMPA 1	2890	protein coding	rs1493383	5	152991398
MSS/MSI-L-OS	HCN1	hyperpolarization activated cyclic nucleotide-gated potassium channel 1	348980	protein coding	rs13180087	5	45265768
MSS/MSI-L-DFS	LINC01121	long intergenic non-protein coding RNA 1121	400952	non-coding RNA	rs6720296	2	45408269
MSS/MSI-L-DFS	n/a (intergenic)	n/a	n/a	n/a	rs1407508	9	101644538
MSS/MSI-L-DFS	n/a (intergenic)	n/a	n/a	n/a	rs912294	13	31698294
colon-OS	n/a (intergenic)	n/a	n/a	n/a	rs4812219	20	59422971
colon-OS	ISM1	isthmin 1, angiogenesis inhibitor	140862	protein coding	rs6105057	20	13229326
colon-OS	ISM1	isthmin 1, angiogenesis inhibitor	140862	protein coding	rs6134830	20	13225350

colon-OS	HCN1	hyperpolarization activated cyclic nucleotide-gated potassium channel 1	348980	protein coding	rs13180087	5	45265768
colon-OS	n/a (intergenic)	n/a	n/a	n/a	rs17325431	23	148462072
colon-OS	ISM1	isthmin 1, angiogenesis inhibitor	140862	protein coding	rs6109769	20	13216257
colon-OS	DSG3	desmoglein 3	1830	protein coding	rs3794924	18	29041734
colon-OS	n/a (intergenic)	n/a	n/a	n/a	rs10921219	1	192587863
colon-OS	n/a (intergenic)	n/a	n/a	n/a	rs10733072	1	192596512
colon-OS	n/a (intergenic)	n/a	n/a	n/a	rs17280262	14	97053924
colon-OS	DPP10	dipeptidyl-peptidase 10	57628	protein-coding (non-functional)	rs17048372	2	115213756
colon-OS	DSG3	desmoglein 3	1830	protein coding	rs8091481	18	29051677
colon-OS	n/a (intergenic)	n/a	n/a	n/a	rs12062810	1	192569804
colon-OS	PARVG	parvin, gamma	64098	protein coding	rs139156	22	44596085
colon-OS	n/a (intergenic)	n/a	n/a	n/a	rs4844011	23	148392640
colon-DFS	n/a (intergenic)	n/a	n/a	n/a	rs17280262	14	97053924
colon-DFS	n/a (intergenic)	n/a	n/a	n/a	rs8035094	15	33564482
colon-DFS	C20orf27	chromosome 20 open reading frame 27	54976	protein coding	rs658495	20	3737495
rectum-OS	n/a (intergenic)	n/a	n/a	n/a	rs17026425	4	150672514
rectum-OS	n/a (intergenic)	n/a	n/a	n/a	rs6854845	4	75746665
rectum-OS	n/a (intergenic)	n/a	n/a	n/a	rs157411	5	67294907
rectum-OS	EFR3A	EFR3 homolog A (S. cerevisiae)	23167	protein coding	rs7004484	8	132934538
rectum-OS	n/a (intergenic)	n/a	n/a	n/a	rs338389	15	68260008
rectum-OS	n/a (intergenic)	n/a	n/a	n/a	rs1555895	10	837407
rectum-OS	n/a (intergenic)	n/a	n/a	n/a	rs16867335	2	181458934
rectum-OS	n/a (intergenic)	n/a	n/a	n/a	rs6739798	2	181465853
rectum-OS	n/a (intergenic)	n/a	n/a	n/a	rs1573948	6	6786425
rectum-OS	n/a (intergenic)	n/a	n/a	n/a	rs10152207	15	38129779
rectum-OS	n/a (intergenic)	n/a	n/a	n/a	rs1827439	15	38123182
rectum-OS	n/a (intergenic)	n/a	n/a	n/a	rs10153021	15	38123785
rectum-DFS	n/a (intergenic)	n/a	n/a	n/a	rs1570271	10	115288501
rectum-DFS	AC011343.1	uncharacterized LOC101927766	101927766	non-coding RNA	rs17057166	5	159248014
rectum-DFS	n/a (intergenic)	n/a	n/a	n/a	rs4868304	5	173131457

rectum-DFS	n/a (intergenic)	n/a	n/a	n/a	rs6854845	4	75746665
rectum-DFS	SLC22A23	solute carrier family 22, member 23	63027	protein coding	rs4959799	6	3295028
rectum-DFS	n/a (intergenic)	n/a	n/a	n/a	rs11138220	9	82060528
rectum-DFS	n/a (intergenic)	n/a	n/a	n/a	rs11138231	9	82066164
rectum-DFS	n/a (intergenic)	n/a	n/a	n/a	rs1015311	9	82078454
rectum-DFS	n/a (intergenic)	n/a	n/a	n/a	rs11138250	9	82083082
rectum-DFS	n/a (intergenic)	n/a	n/a	n/a	rs10491791	9	82096194
rectum-DFS	n/a (intergenic)	n/a	n/a	n/a	rs13286432	9	82098952
rectum-DFS	n/a (intergenic)	n/a	n/a	n/a	rs1890836	9	82119180
rectum-DFS	*n/a (intergenic)	n/a	n/a	n/a	rs10275272	7	19160897
rectum-DFS	ANO1	anoctamin 1, calcium activated chloride channel	55107	protein coding	rs3781663	11	69999252
rectum-DFS	n/a (intergenic)	n/a	n/a	n/a	rs9419702	10	133531153
rectum-DFS	n/a (intergenic)	n/a	n/a	n/a	rs11138252	9	82087111

Chr: chromosome, DFS: disease-free survival, MSS: microsatellite stable, MSI-L: microsatellite instability-low, n/a: not available, OS: overall survival, SNP: single nucleotide polymorphism. *close to a gene.

References

(1) Sherry ST, Ward MH, Kholodov M, Baker J, Phan L, Smigielski EM, et al. dbSNP: the NCBI database of genetic variation. Nucleic Acids Res 2001 Jan 1;29(1):308-311.

(2) Maglott D, Ostell J, Pruitt KD, Tatusova T. Entrez Gene: gene-centered information at NCBI. Nucleic Acids Res 2011 Jan;39(Database issue):D52-7.
