# Supplementary material for: A genome wide association study on Newfoundland colorectal cancer patients’ survival outcomes
Source: Biomark Res. 2015 Mar 19;3:6. doi: 10.1186/s40364-015-0031-6 (PMC4393623; doi:10.1186/s40364-015-0031-6)
Supplement: Additional file 5: — Principal Component Analysis (PCA) and Multidimensional Scaling (MDS) analysis results for the patient cohort. [file 40364_2015_31_MOESM5_ESM.pdf]

Additional File 5. Principal Component Analysis (PCA) and Multidimensional Scaling (MDS) analysis results for the patient cohort

a) MDS plots

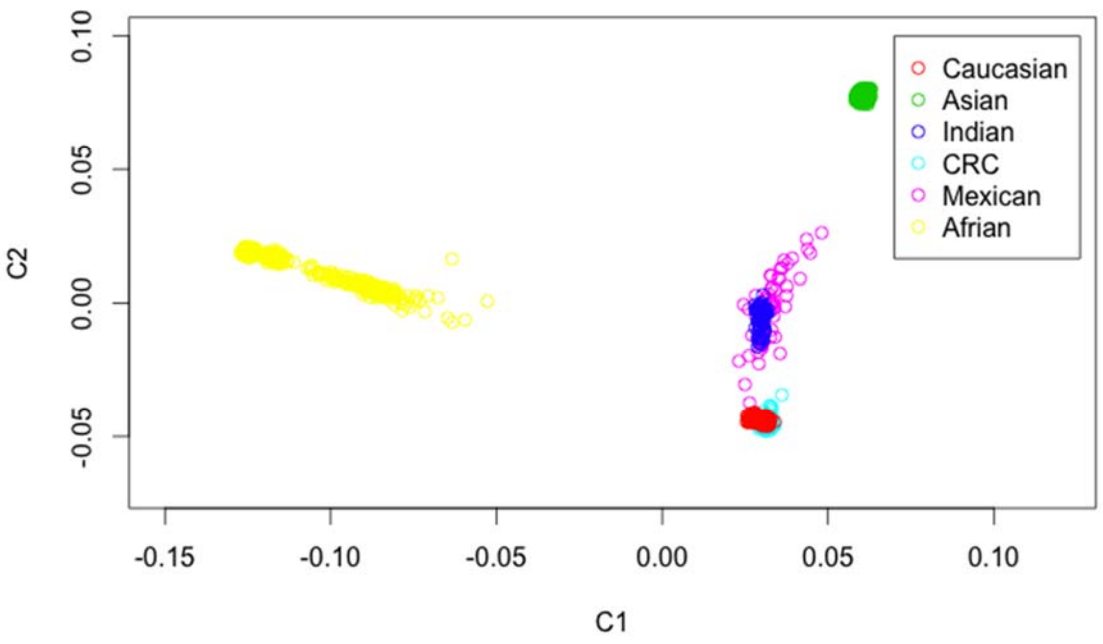

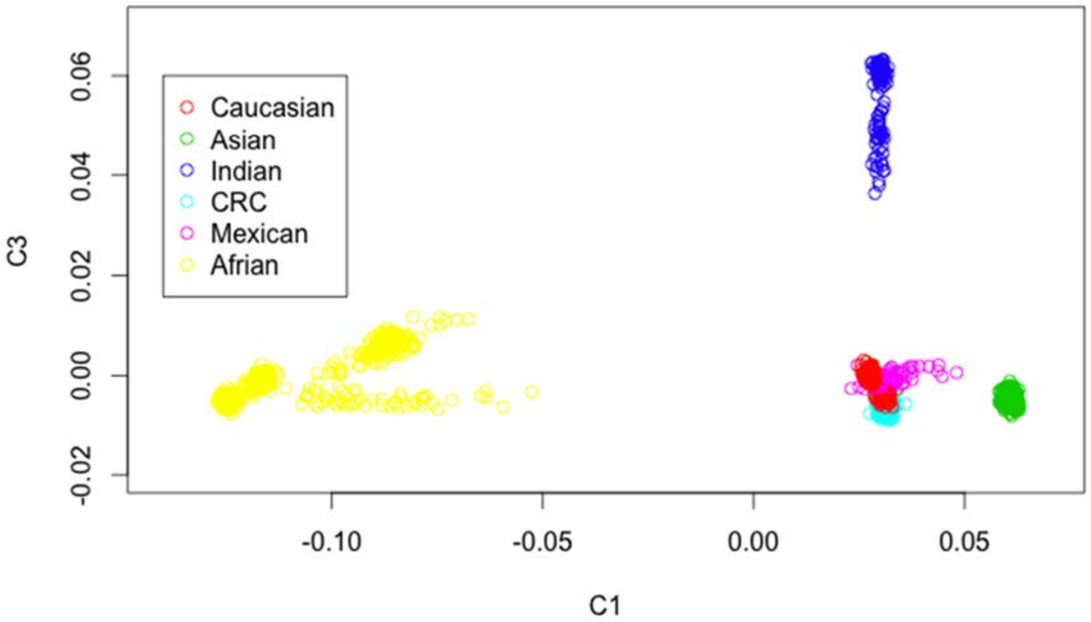
Caucasian
Asian
Indian
CRC
Mexican
Afrian
C3
0.06
0.04
0.02
0.00
-0.02
-0.10
-0.05
0.00
0.05
C1

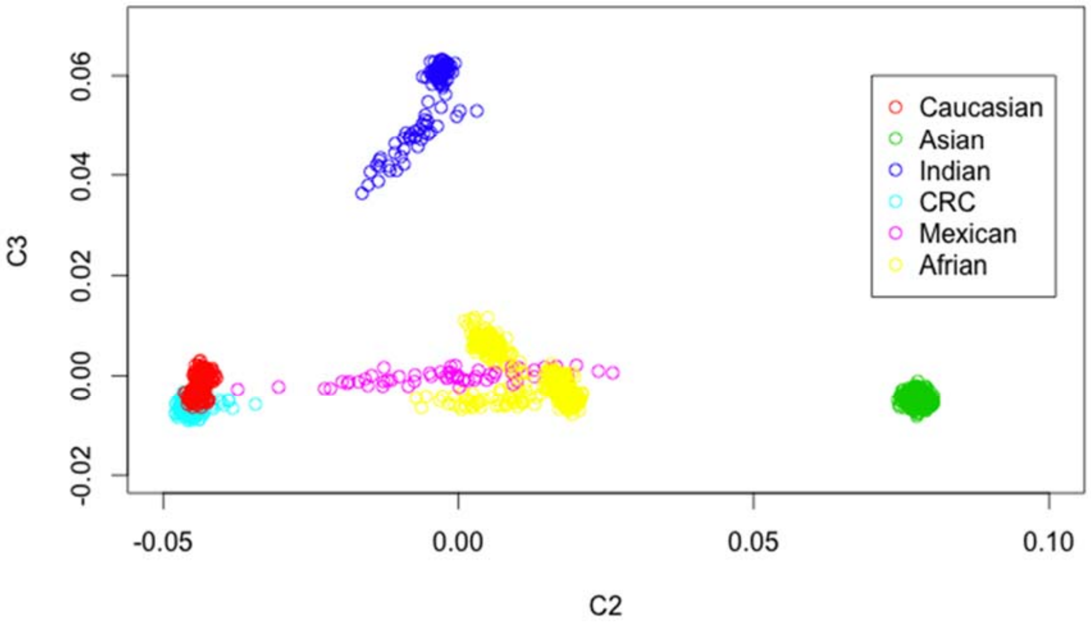
Caucasian
Asian
Indian
CRC
Mexican
Afrian
C3
0.06
0.04
0.02
0.00
-0.02
-0.05
0.00
0.05
0.10
C2

Multidimensional-scaling (MDS) is used to identify individuals with diversity in ancestry. The three plots attached here show that the patients recruited in our study, labelled as “CRC”, are quite close to the cluster of Caucasians in the graphs.

b) Eigenvalues from PCA

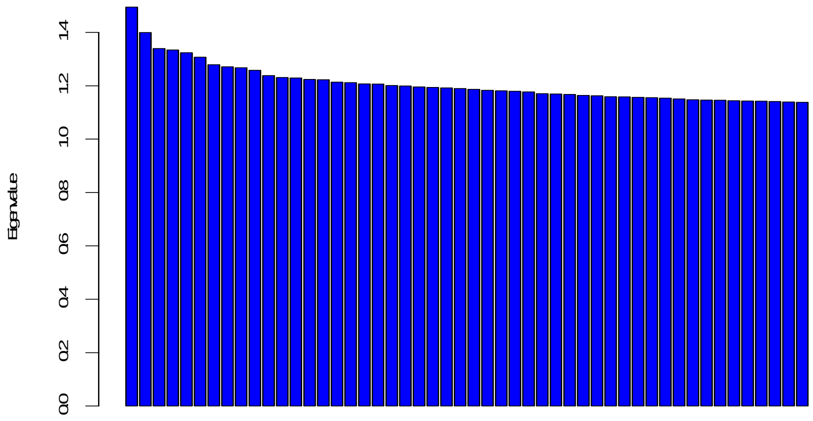

Eigenvalues extracted from PCA can be used to explain the variance of genetic information involved in our data in a lower dimension. Some extremely high eigenvalues reflect the existence of ancestry difference within the data, otherwise population stratification does not need to be considered.
